# Nuclear HMGB1 Loss in the Aging Brain Triggers cGAS‐STING Pathway and Neuroinflammation that Contributes to Senescence and Cognitive Decline in Tauopathies

**DOI:** 10.1002/alz.089521

**Published:** 2025-01-03

**Authors:** Sagar Gaikwad, Md Anzarul Haque, Madison Samples, Rhea Xavier, Cynthia Jerez, Fadhl Al‐Shaebi, Rakez Kayed

**Affiliations:** ^1^ University of Texas Medical Branch, Galveston, TX USA; ^2^ UTMB, Galveston, TX USA; ^3^ utmb galveston, GALVESTON, TX USA

## Abstract

**Background:**

Aging, tau pathology, and chronic inflammation in the brain play crucial roles in neuroinflammation, synaptic loss, neurodegeneration, and cognitive decline in tauopathies, such as Alzheimer’s disease. However, the molecular mechanisms that trigger aberrant chronic inflammatory signaling in tauopathies are poorly understood.

**Method:**

We utilized brain tissues from tauopathy patients and the tauopathy mouse models. Our study involved immunofluorescence microscopy and biochemical assays to investigate the role of nuclear HMGB1 in DNA damage, brain inflammation, senescence, and cognitive dysfunction. We evaluated impact of HMGB1 knockdown on tau pathology (including phospho‐tau, tau oligomers, and neurofibrillary tangles), senescence, inflammation, synaptic and postsynaptic proteins, as well as cognitive functions using the Y‐maze and novel‐object recognition tests.

**Result:**

Here, we show that nuclear high mobility group box 1 (HMGB1) protein is reduced in the brains of human tauopathy patients and mouse models of tauopathies. Knockdown of nuclear HMGB1 using AAV‐based shRNA resulted in increased DNA‐damage and activation of the cyclic GMP–AMP synthase stimulator of interferon genesis (cGAS–STING) signaling pathway in the brain of hTau tauopathy mice. The activation of cGAS–STING upon HMGB1 knockdown induced inflammatory phenotypes of senescent cells and promoted neuroinflammation, tau pathology, synaptic loss, and cognitive decline in hTau mice. Further investigations in human tauopathy brains revealed that HMGB1 loss enhances DNA damage, and cytosolic DNA release, activating cGAS signaling and bystander inflammation.

**Conclusion:**

Our findings demonstrate that during tauopathies, the loss of nuclear HMGB1 leads to DNA‐damage and triggers activation of the cGAS–STING pathway, contributing to aging‐related inflammation, tau pathology and synaptic loss in the brain, and ultimately leading to cognitive decline.

